# Advancing rare disease treatment: EMA’s decade-long insights into engineered adoptive cell therapy for rare cancers and orphan designation

**DOI:** 10.1038/s41434-024-00446-0

**Published:** 2024-03-14

**Authors:** Maria Elisabeth Kalland, Tomas Pose-Boirazian, Gloria Maria Palomo, Frauke Naumann-Winter, Enrico Costa, Darius Matusevicius, Dinah M. Duarte, Eva Malikova, Dinko Vitezic, Kristina Larsson, Armando Magrelli, Violeta Stoyanova-Beninska, Segundo Mariz

**Affiliations:** 1 Norwegian Medical Products Agency, Grensesvingen 26, 0663 Oslo, Norway; 2 Committee for Orphan Medicinal Products, European Medicines Agency, Domenico Scarlattilaan 6, 1083 HS Amsterdam, The Netherlands; 3 Orphan Medicines Office, European Medicines Agency, Domenico Scarlattilaan 6, 1083 HS Amsterdam, The Netherlands; 4 Agencia Española de Medicamentos y Productos Sanitarios, Calle Campezo n° 1, Edificio 8, 28022 Madrid, Spain; 5 Bundesinstitut für Arzneimittel und Medizinprodukte, Kurt-Georg-Kiesinger-Allee 3, 53175 Bonn, Germany; 6 Agenzia Italiana del Farmaco, Via del Tritone, 181, 00187 Rome, Italy; 7 Swedish Medical Products Agency, Dag Hammarskjölds väg 42, 752 37 Uppsala, Sweden; 8 INFARMED - National Authority of Medicines and Health Products, I.P., Avenida do Brasil 53, 1749-004 Lisbon, Portugal; 9 Universidade de Lisboa, Faculdade de Farmácia, Avenida Professor Gama Pinto, 1649-003 Lisbon, Portugal; 10 State Institute for Drug Control, Kvetná 11, 825 08 Bratislava, Slovakia; 11 Department of Pharmacology and Toxicology, Comenius University, Odbojárov 10, 832 32 Bratislava, Slovakia; 12 University of Rijeka, Faculty of Medicine, and University Hospital Centre Rijeka, Braće Branchetta 20/1, 51000 Rijeka, Croatia; 13 National Center for Drug Research and Evaluation, Istituto Superiore di Sanità, Viale Regina Elena, 299, 00161 Rome, Italy; 14 College ter Beoordeling van Geneesmiddelen, Graadt van Roggenweg 500, 3531 AH Utrecht, The Netherlands

**Keywords:** Haematological cancer, Cell therapies, Biomarkers, Immunotherapy

## Abstract

Adoptive cell therapy (ACT), particularly chimeric antigen receptor (CAR)-T cell therapy, has emerged as a promising approach for targeting and treating rare oncological conditions. The orphan medicinal product designation by the European Union (EU) plays a crucial role in promoting development of medicines for rare conditions according to the EU Orphan Regulation.

This regulatory landscape analysis examines the evolution, regulatory challenges, and clinical outcomes of genetically engineered ACT, with a focus on CAR-T cell therapies, based on the European Medicines Agency’s Committee for Orphan Medicinal Products review of applications evaluated for orphan designation and maintenance of the status over a 10-year period. In total, 30 of 36 applications were granted an orphan status, and 14 subsequently applied for maintenance of the status at time of marketing authorisation or extension of indication. Most of the products were autologous cell therapies using a lentiviral vector and were developed for the treatment of rare haematological B-cell malignancies. The findings revealed that 80% (29/36) of the submissions for orphan designation were supported by preliminary clinical data showing a potential efficacy of the candidate products and an added clinical benefit over currently authorised medicines for the proposed orphan condition. Notably, in 89% (32/36) of the cases significant benefit of the new products was accepted based on a clinically relevant advantage over existing therapies. Twelve of fourteen submissions reviewed for maintenance of the status at time of marketing authorisation or extension of indication demonstrated significant benefit of the products over existing satisfactory methods of treatment within the approved therapeutic indications, but one of the applications was withdrawn during the regulatory evaluation.

This article summarises the key findings related to the use of engineered ACT, primarily CAR-T cell therapies, in targeting and treating rare cancers in the EU. It emphasises the importance of use of clinical data in supporting medical plausibility and significant benefit at the stage of orphan designation and highlights the high success rate for these products in obtaining initial orphan designations and subsequent maintaining the status at the time of marketing authorisation or extension of indication.

## Introduction

Adoptive cell therapy (ACT) is a type of cancer immunotherapy that exploits the body’s immune system to enhance anti-tumour activity and overall immunity to combat cancer. It involves transfer of immune cells into patients and has shown promising results in the treatment of various tumour types, with high response rates and even durable remissions in some cases, particularly haematological malignancies [1–5]. However, there are still numerous challenges to overcome, such as managing potential serious side effects and improving the effectiveness of ACT in solid tumours [6, 7]. The technique is mainly based on the concept that the immune system can control a patient’s cancer in the long-term and has been demonstrated through three independent main approaches [8].

The first approach involves use of tumour-infiltrating lymphocytes (TIL), which can be isolated from a patient’s tumour lesions, expanded in the laboratory, and then reinfused back into the patient to target and kill cancer cells [8]. The second approach comprises use of genetically engineered T-cells that are modified in the laboratory to express modified T-cell receptors (TCR) that can recognise cancer-specific antigens, making them capable of inducing tumour regressions upon re-infusion into the patient [8, 9]. The third approach are chimeric antigen receptor (CAR)-engineered T-cells that are designed to express synthetic receptors that have the specificity of a monoclonal antibody and signalling domains capable of inducing a cascade of events in the CAR-engineered immune cells upon target engagement, which allows them to recognise, target, and destroy specific types of cancer cells.

Genetically modified T-cells with engineered CAR or TCR as a group represent a unique approach in the treatment of rare cancers and are the focus of this article. The Committee for Orphan Medicinal Products (COMP) at the European Medicines Agency (EMA) helps to incentivize the development of these therapies through the orphan medicinal product designation process, which is regulated by the Orphan Regulation in the EU [10, 11]. The COMP has seen this field evolve over the years through the submissions received for orphan designation (OD) and maintenance of the status. The first CAR-T cell product to apply for an OD was designated in 2014. The COMP has hence assessed OD applications for CAR-T cell products over the past decade, with some of these products reaching the marketing authorisation (MA) application stage. In fact, the first two CAR-T cell products (Yescarta and Kymriah) with MA in the EU from 2018 had an OD and are approved as orphan medicinal products. These medicines are advanced therapy medicinal products (ATMP) according to the European legislation [12].

The COMP assesses applications for a product at two different stages. An initial OD submission is one where the COMP determines three main criteria. Firstly, the proposed condition needs to fulfil the definition of an orphan condition, which means that it is a well-defined medical entity documented in the public domain, chronically debilitating and/or life-threatening, and has a prevalence below 5 in 10,000 [13, 14]. Secondly, the product must show a promise that it could be effective in treating the condition, by establishing medical plausibility (MP) that can be defined as a demonstration of the “intention to treat”, to support the rationale for the development of the product in the proposed condition and for the assumption of a clinical benefit [15]. Finally, where satisfactory methods such as authorised medicines exist for the orphan condition [16], it must be shown that the product offer a potential significant benefit (SB) to these methods [17, 18]. The SB criterion is unique to the EU Orphan Regulation framework. ‘Significant benefit’ is defined in Article 3(2) of Regulation (EC) No 847/2000 as ‘a clinically relevant advantage or a major contribution to patient care’ [11]. ‘A clinically relevant advantage’ may be based on improved efficacy for the entire population suffering from the condition or a particular population subset or a subset that is resistant to the existing treatments, or a better safety profile or a better tolerability for the entire population suffering from the condition or for a particular subset [19]. ‘A major contribution to patient care’ may be based on ease of self-administration, e.g., if the new treatment allows ambulatory treatment instead of treatment in a hospital only or if it has a significant impact on convenience of use and reduces treatment burden, or significantly improved adherence to treatment due to a change in pharmaceutical form (e.g., modified release formulation) [19].

The second assessment occurs at the time of a parallel submission for a MA or MA extension of the product and is called the review of the OD criteria for the maintenance of the status. At this stage, the COMP reviews the rareness of the condition again using the same criteria as for the initial OD. MP is not assessed, as it is considered established based on a positive benefit-risk assessment by the Committee for Medicinal Products for Human Use (CHMP). SB is assessed again and requires a review of the satisfactory methods available at that time point as they may have changed since the OD was granted. The COMP will expect the applicant to provide confirmatory data to support SB and to present data supporting the claim for SB of the candidate product within the context of the treatment algorithm considering all currently valid satisfactory methods [16].

In this analysis, we reviewed the evaluation of all applications regarding genetically modified T-cells with engineered CAR or TCR submitted to the COMP seeking OD or maintenance of the status over the past ten years and analysed the regulatory decision-making based on the data provided. This article aims to communicate the findings primarily associated with the most submitted genetically engineered ACT, namely CAR-T cell therapy. It details the type of data used to support MP of the candidate products and SB over existing satisfactory methods in the proposed orphan condition at the initial designation stage. The analysis also explores the impact of data used to support SB at the time of review of the OD criteria for maintenance of the status which occurs at MA or extension of indication. This is an important factor in the decision to recommend granting the 10-year market exclusivity for medicines approved as orphan medicinal products. By sharing information about the regulatory process and outcomes of OD applications, it is hoped that it will provide valuable insights for stakeholders involved in the development and regulation of these therapies and help future sponsors to have a better understanding of some of the success factors associated with the OD and maintenance of the status to advance this rapidly evolving therapeutic field.

## Materials and methods

Summary reports of the applications for OD (*n* = 36) and those for the maintenance of OD at the time of licensing (*n* = 14) for genetically engineered ACT concluded by the COMP/ European Commission (EC) between 1st of January 2014 and 31st of December 2023 were screened for information about the number received and outcome of the applications (positive, negative, or withdrawn), year of opinion/ designation date, current OD status (active or withdrawn), the origin and type of sponsor (applicant) both at the designation stage and at time of MA/ MA extension, and eventual transfer of the OD to another sponsor. Data extraction also focused on type of product (CAR-T cells versus TCR-redirected T-cells) and (non-)viral vector used to produce the genetically engineered ACT, and the target antigen. Information was collected regarding the targeted orphan condition, its prevalence, evidence used to support MP and the assumption of SB over existing therapies at the initial OD stage. Additionally, as six of these products have already obtained a MA, data used to demonstrate SB within the targeted therapeutic indications and confirm the OD criteria at the time of review for the 14 applications received for maintenance of the status were collected.

### Information about the type of product

Data collected focused on type of product which included purified T-cell type, (non-)viral vector used, and antigen targeted.

### Data provided to support MP

The data submitted and used to support MP was categorised into non-clinical in vitro or in vivo data and preliminary clinical study data. Each of these sections was further subdivided as summarised below.

#### Non-clinical in vitro or in vivo data

The non-clinical in vitro or in vivo data presented and accepted as evidence in the submissions were collected. The validity of the non-clinical models used for recapitulating the aspects of the pathophysiology of the condition, and eventually use of surrogate product in these models were recorded. Additionally, for in vivo data, phenotypic/disease as well as surrogate-relevant endpoints linked to the condition were defined and collected. These involved biomarkers which were specific to the condition and survival principally.

#### Preliminary clinical study data

OD applications which had clinical study data were entered into the database. Clinical data was categorised by the type of study they were obtained from such as a phase 1, 2, or 3, or investigator-initiated study as well as other sources such as case reports or compassionate use programme, number of patients evaluated, and clinical endpoints used to show efficacy of the product. Occasionally published data was used by sponsors in submissions and was collected.

### Data provided to support SB

Data collected for SB for initial OD was focused on submissions where other satisfactory methods were available for the target patient population. Additionally, fulfilment of this criterion and the evidence accepted to support an assumption of SB was noted. The category of data presented was further subdivided into claims of SB over either other therapies or other ATMP/innovative therapies based on (1) a CRA, (2) a major contribution to patient care, or (3) both CRA and major contribution to patient care.

Data used to demonstrate SB within the proposed therapeutic indication at the time of MA or extension of indication for the review of the criteria for the maintenance of the OD was collected using the same criteria as for an initial OD.

## Results

A total of 23 ACT with genetically modified peripheral blood T-cells were identified in the 36 applications received for an OD (Table 1). Of these, 19 (83%) were CAR-T cell products and 4 (17%) were TCR-modified T-cell products. Overall, 19 of the 23 genetically engineered ACT (83%) were granted at least one designation covering 30 different OD. Four products were included in more than one OD application for the treatment of different orphan conditions and have been granted MA for some of them. These were all cluster of differentiation 19 (CD19)-directed autologous CAR-T cell products using either lentiviral- or retroviral vectors developed to treat patients with rare B-cell leukaemia and/or lymphoma. The sponsors of these four CAR-T cell products applied for either up to two, three, four, or eight different OD for each product (Table 1). For two of these products, one OD application each were withdrawn during the regulatory evaluation before an opinion was adopted. An additional two autologous CAR-T cell products using a lentiviral vector and targeting the B-cell maturation antigen (BCMA) were designated for the treatment of multiple myeloma and have been granted a conditional MA.

**Table 1:** Overview of applications for OD and maintenance of OD at the time of MA evaluated by the COMP for genetically engineered ACT in the period from 2014 to 2023.

A The OD status for the submissions for six autologous CAR-T cell products which have reached the MA stage and their MA status.									
#	Product name/ INN (Product code)	Purified cells	(non-)viral vector	Target antigen	Outcome of OD application	Current OD status	Orphan conditions	MA	Approved indication (year)
**1**	Abecma/ idecabtagene vicleucel (bb2121)	CD3	Lentivirus	BCMA	Positive	Active	MM	CMA	4 L + RRMM (2021)
**2**	Carvykti/ ciltacabtagene autoleucel (JNJ-68284528)	CD3	Lentivirus	BCMA	Positive	Active	MM	CMA	4 L + RRMM (2022)
**3**	Kymriah/ tisagenlecleucel (CTL019)	CD3	Lentivirus	CD19	Positive	Active	ALL, DLBCL, FL	MA/EoI	3 L + DLBCL, r/r B-cell ALL (2018), 3 L + FL (2022)
					Withdrawn	N/A	CLL/SLL	No	N/A
**4**	Breyanzi/ lisocabtagene maraleucel (JCAR017)	CD4, CD8	Lentivirus	CD19	Positive	Withdrawn	DLBCL, FL, PMBCL	MA	3 L + DLBCL, PMBCL, FL grade 3B (2022)
**5**	Yescarta/ axicabtagene ciloleucel (KTE-C19)	CD3	Retrovirus	CD19	Positive	Active	DLBCL, FL, MZL, PMBCL	MA/EoI	3 L + DLBCL, PMBCL (2018), 4 L + FL (2022), 2 L DLBCL (2022)
						Withdrawn	ALL, CLL/SLL, MCL	No	N/A
					Withdrawn	N/A	HGBCL	No	N/A
**6**	Tecartus/ brexucabtagene autoleucel (KTE-X19)	CD4, CD8	Retrovirus	CD19	Positive	Active	MCL, ALL	CMA/EoI	3 L + MCL (2020), adult r/r B-cell ALL (2022)

B The OD status for the submissions for 13 CAR-T cell products and 4 TCR-modified T-cell products which have been assessed but not reached the MA stage.							
#	Active substance (Product code)	Purified cells	(non-)viral vector	Target antigen	Outcome of OD application	Current OD status	Orphan conditions
**CAR-T cell therapy**							
	***Autologous CAR-T cells***						
**1**	Autologous T lymphocyte-enriched population of cells transduced with a lentiviral vector encoding a CAR targeting human BCMA with 4-1BB and CD3-zeta intracellular signalling domains (CT053)	CD3	Lentivirus	BCMA	Positive	Active	MM
**2**	Orvacabtagene autoleucel (JCARH125)	CD4, CD8	Lentivirus	BCMA	Positive	Withdrawn	MM
**3**	Autologous T-cells transduced with lentiviral vector encoding an anti-SLAMF7 CD28/CD3-zeta CAR	CD4, CD8	Lentivirus	SLAMF7	Positive	Active	MM
**4**	Autologous T cells ex vivo modified with a lentiviral vector encoding a CAR specific for CD1a (OC-1)	CD3	Lentivirus	CD1a	Positive	Active	ALL
**5**	Autologous T-cells transduced with a lentiviral vector encoding a CAR against CD7 (PA3-17)	CD3	Lentivirus	CD7	Positive	Active	ALL
**6**	Obecabtagene autoleucel (AUTO1)	CD3	Lentivirus	CD19	Positive	Active	ALL
**7**	Autologous T cells transduced with lentiviral vector containing a tandem CAR directed against CD20 and CD19 (MB-CART2019.1)	CD4, CD8	Lentivirus	CD20/CD19	Positive	Active	DLBCL
**8**	Autologous T lymphocyte-enriched population of cells transduced with a lentiviral vector encoding a CAR targeting human claudin 18.2 antigen with CD28 and CD3-zeta intracellular signalling domains (CT041)	CD3	Lentivirus	CLDN18.2	Positive	Active	GC
**9**	Autologous T cells transduced with lentiviral vector containing a CAR directed against CD123	CD3	Lentivirus	CD123 (IL-3R)	Positive	Active	BPDCN
**10**	Autologous T cells transduced with a lentiviral vector expressing a CAR against CLL-1 (BG1805)	CD3	Lentivirus	CLL-1 (CD371)	Positive	Active	AML
	***Allogenic CAR-T cells***						
**11**	Allogeneic CRISPR/Cas9-mediated genetically modified CAR T cells targeting CD19 antigen (CTX110)	CD3	AAV	CD19	Withdrawn	N/A	DLBCL
	***Autologous CAR-T regulatory cells***						
**12**	Autologous naive regulatory T cells transduced with a lentiviral vector encoding for a CAR to recognise the HLA-A*02 antigen (TX200-TR101)	CD4	Lentivirus	HLA-A2	Positive	Active	SOT
**13**	Autologous T regulatory cells expressing an HLA-A2-specific CAR (QEL-001)	CD4	Lentivirus	HLA-A2	Withdrawn	N/A	SOT
**TCR-based therapy**							
	***Autologous TCR-modified T-cells***						
**14**	Autologous CD4+ and CD8 + T cells transduced with a lentiviral vector encoding an affinity enhanced TCR specific to MAGE-A4 (ADP-A2M4)	CD4, CD8	Lentivirus	MAGE-A4	Positive	Active	STS
**15**	Autologous CD4+ and CD8 + T-cells transduced with lentiviral vector containing an affinity-enhanced TCR targeting the New York oesophageal antigen-1 (NY-ESO-1c259T)	CD4, CD8	Lentivirus	NY-ESO-1	Positive	Active	STS
**16**	Autologous T cells transduced with retroviral vector encoding a hepatitis B virus antigen-specific TCR (LioCyx-D)	CD4, CD8	Retrovirus	HBV	Withdrawn	N/A	HC
**17**	Autologous T cells transfected with mRNA encoding hepatitis B virus antigen-specific TCR (LioCyx-M)	CD4, CD8	messenger RNA	HBV	Withdrawn	N/A	HC

*AAV* adeno-associated vector, *ALL* Acute lymphoblastic leukaemia, *BCMA* B-cell maturation antigen, *BPDCN* Blastic plasmacytoid dendritic cell neoplasm, *CAR* chimeric antigen receptor, *CD* cluster of differentiation, *CLL-1* C-type lectin-like molecule-1, *CLL/SLL* Chronic lymphocytic leukaemia/ small lymphocytic lymphoma, *CMA* conditional marketing authorisation, *DLBCL* Diffuse large B-cell lymphoma, *EoI* extension of indication, *FL* Follicular lymphoma, *GC* Gastric cancer, *HBV* hepatitis B virus, *HC* Hepatocellular carcinoma, *HGBCL* High-grade B-cell lymphoma, *HLA* human leucocyte antigen, *IL-3R* interleukin-3 receptor, *MA* marketing authorisation, *MAGE-A4* melanoma-associated antigen family A4, *MCL* Mantle cell lymphoma, *MZL* Marginal zone lymphoma, *MM* Multiple myeloma, *N/A* not applicable, *NY-ESO-1* New York oesophageal antigen-1, *PMBCL* Primary mediastinal large B-cell lymphoma, *r/r* relapsed or refractory, *RRMM* relapsed and refractory multiple myeloma, *SLAMF7* signalling lymphocytic activation molecule F7, *SOT* Solid organ transplantation, *STS* Soft tissue sarcoma, *TCR* T-cell receptor, *2* *L* second-line therapy, *3* *L+* third- and later lines of therapy, *4* *L+* fourth- and later lines of therapy.

In total, six (17%) of the OD applications for six products were withdrawn due to insufficient data to fulfil the criteria for an OD. The reasons for the withdrawals were related to (1) failure to provide reliable prevalence estimates based on relevant epidemiologic data sources that showed that the condition (i.e., CLL/SLL) did not breach the prevalence threshold required for an orphan status (1 application), (2) the proposed orphan condition (i.e., HGBCL) was not considered a distinct medical entity, but rather a subtype of a broader condition for which the product already had an OD (1 application), and (3) failure to provide sufficient data to justify the assumption of SB over existing therapies for the proposed conditions (4 applications). It was also noted that the sponsors for three (16%) of the 19 products that were granted either one or several OD withdrew the orphan status for some of the designated conditions, which constituted seven OD in total.

Eighteen (78%) of the products evaluated for OD used a lentiviral vector (16 CAR-T cell products, 2 TCR-modified T-cell products). Only three products used a retrovirus vector (2 CAR-T cell products, 1 TCR-modified T-cell product), one CAR-T cell product used an adeno-associated vector, and one TCR-modified T cell product used messenger RNA. A variety of target antigens were selected for these products with the most common being CD19, which is widely expressed on malignant B-cells, representing 7 (30%) of the 23 products in total, including one bispecific CAR-T cell product targeting both CD19 and CD20. Another common target for 4 (17%) of the products was BCMA, which is expressed on the cell surface of myeloma cells, normal plasma cells, and a small subset of normal B-cells. Other antigens targeted by the genetically engineered ACT included: (2) human leucocyte antigen (HLA)-A2, (2) hepatitis B virus (HBV), (1) signalling lymphocytic activation molecule F7 (SLAMF7), (1) CD1a, (1) CD7, (1) CLDN18.2, (1) CD123 (IL-3R), (1) C-type lectin-like molecule-1 (CLL-1) (CD371), (1) melanoma-associated antigen family A4 (MAGE-A4), and (1) New York oesophageal antigen-1 (NY-ESO-1). The largest group of orphan conditions applied for were haematological conditions, and rare haematological malignancies were designated in 26 (87%) of the 30 OD that were granted, and only 4 OD were given to 4 different products intended to treat either soft tissue sarcoma (2 OD), gastric cancer (1 OD), or solid organ transplantation (1 OD).

There are currently 6 CAR-T cell products approved on the EU market. All six products received both OD and Priority Medicine (PRIME) designations from the COMP/ EMA before the applications for MA were submitted. There were 6 initial MA approved for 10 therapeutic indications, covering 10 different OD, and 4 extensions of the approved indications, covering 3 additional OD (as 1 indication extension was for an earlier treatment line). Three of the authorised CAR-T cell products, i.e., Yescarta, Kymriah, and Breyanzi, received a full approval at the initial MA stage, whereas the other three, i.e., Tecartus, Abecma, and Carvykti, initially received a conditional MA (Table 1A). Conditional MA is recommended by the CHMP when the medicine addresses an unmet medical need and a positive benefit-risk balance for the therapeutic indication is demonstrated, but more comprehensive clinical data to support it in the future is required [20]. All designated products for which maintenance of the OD at the stage of MA or MA extension was applied were developed to treat rare haematological malignancies. None of the two TCR-modified T-cell therapies that were designated for the treatment of soft tissue sarcoma have reached the stage of MA and hence the review of the OD criteria for maintenance of the status.

The most common prevalence of the orphan conditions targeted for in the 36 OD applications received for engineered ACT was estimated to ≥ 3 in 10,000 people in the EU (15/36; 42%), which included multiple myeloma, diffuse large B-cell lymphoma, and follicular lymphoma. The smallest group (9/36; 25%) constituted the orphan conditions with a prevalence of less than 1 in 10,000 people. In total, 12 OD applications (33%) were received for genetically engineered ACT targeting orphan conditions with an estimated prevalence of 1 to < 3 in 10,000 persons. In line with the OD applications, the most common prevalence of the orphan conditions targeted for in the orphan maintenance procedure was ≥ 3 in 10,000 persons (9/14; 64%). For 57% (8/14) of the submissions for maintenance of OD, the prevalence of the targeted orphan conditions had increased since their OD were granted, and the majority of these (6/8) were conditions estimated to be affecting ≥ 3 in 10,000 people in the community.

Large pharmaceutical companies represented the highest number of applicants for OD (22/36; 61%) with small and medium-sized enterprises (SME) and consultancies next (6/36; 17% each). Two OD applications (5%) submitted by each of these three groups of applicants were withdrawn during the regulatory assessment before an opinion was adopted. Only 2 applications for OD (5%) were submitted by academia/charity, and both were granted an OD. In total, 6/30 (20%) granted OD were transferred to another sponsor later. All sponsors that applied for orphan maintenance at the stage of MA or MA extension were large pharmaceutical companies.

### Data used to support MP at the initial OD stage

**Fig. 1:**
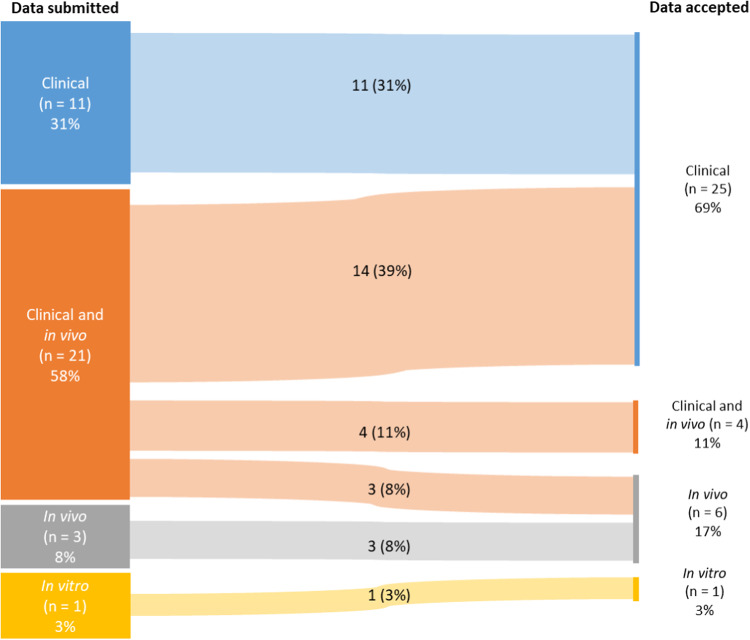
Types of data submitted (left) versus those accepted (right) to support the MP criterion in OD applications received for genetically engineered ACT (*N* = 36). The number of OD applications that included specific types of data and the proportion of these are given in each coloured box. Data derived from clinical studies is marked in blue, data from both clinical- and non-clinical in vivo studies is marked in orange, data based on non-clinical in vivo studies alone is marked in grey, and pure in vitro data is marked in yellow.

Overall, preliminary clinical data was used to support an assumption of MP for the proposed product in 29/36 (81%) OD submissions received for genetically engineered ACT over a 10-years period (Fig. 1). In 25/36 (69%) cases a decision was based purely on clinical data and in 4/36 (11%) on clinical and supporting in vivo data, while in 6/36 (17%) cases the decisions were based only on non-clinical in vivo data. For half of the 6 OD applications where non-clinical in vivo data was the basis for supporting MP, clinical data was also submitted, but considered insufficient for assessment due to limited number of patients. Finally, in 1 submission for OD only in vitro data was used to support MP of the proposed product (Fig. 1).

**Fig. 2:**
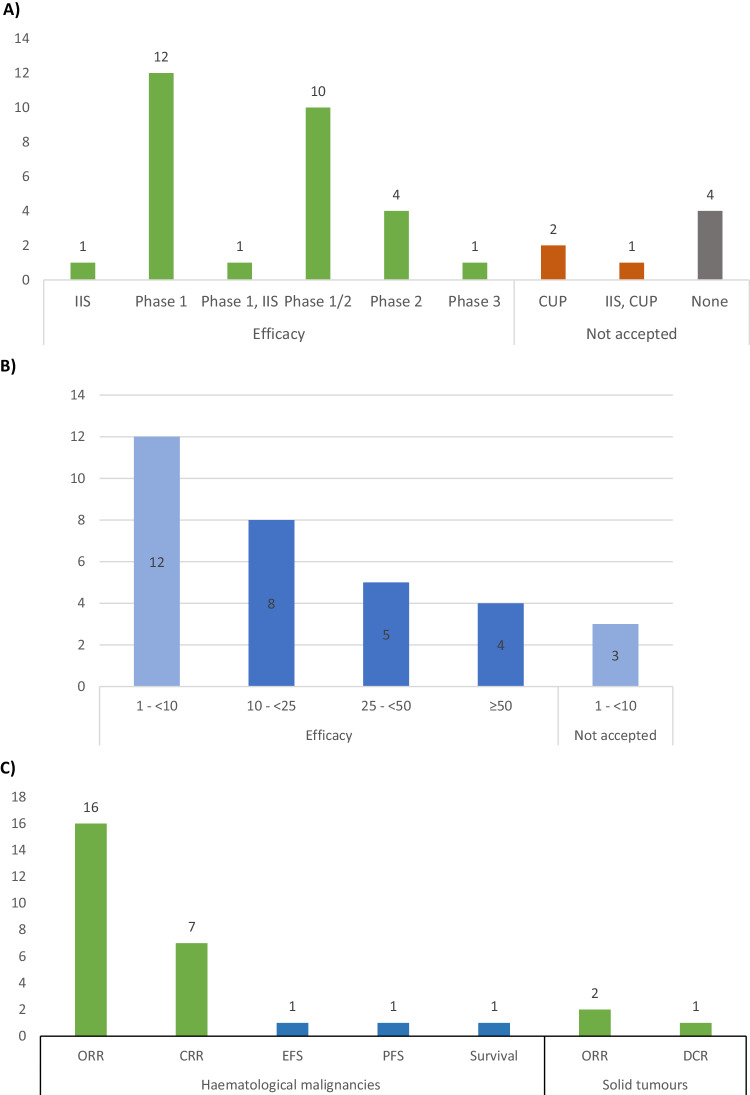
Clinical data used to support an assumption of MP for OD applications. **A** Overview of cases where clinical data were submitted and those accepted as evidence to support MP and the studies the data were collected from (*N* = 36). **B** Summary of numbers of patients, divided into four main categories, which were evaluated in the clinical data provided in the submissions and those accepted as evidence for efficacy to support MP (*n* = 32). **C** Overview of the clinical endpoints accepted to support MP in the submissions excluding those that were not supported by clinical data (*n* = 29). CRR complete response rate, CUP compassionate use programme, DCR disease-control rate (including ORR and stable disease), EFS event-free survival, IIS Investigator-initiated study, None No clinical data was provided in the submissions, ORR objective/overall response rate, PFS progression-free survival.

The preliminary clinical data used to support the MP criterion for OD was mainly obtained from single-arm phase 1 or phase 1/2 studies (23/29; 79%) (Fig. 2A). Most of the clinical data in the submissions were collected from small patient populations of < 25 patients (23/32; 72%), but some included data based on observations in populations of ≥ 50 patients (4 applications) (Fig. 2B). Notably, 3 OD applications with clinical data from only one or two patients in compassionate use programmes, and a few additional patients from an investigator-initiated study for one of them, failed to show MP in the proposed orphan conditions. The most common clinical endpoints used to support a potential efficacy of the products were objective anti-tumour responses in terms of overall- (ORR) and complete response rates (CRR) (25/29; 86%) (Fig. 2C).

The non-clinical in vivo data used to show potential efficacy of the candidate products to support MP were derived from valid mouse models of the proposed orphan conditions where the tumour burden and survival of treated mice were evaluated. In all OD applications where these data were accepted (*n* = 10; 28%), the in vivo data showed reduced tumour burden and prolonged survival of the mice treated with the candidate- or murine surrogate products over control treatments. In vivo tumour xenograft mouse models using non-obese diabetic severe combined immune deficiency IL-2RG (null) (NOD-SCID-gamma null; NSG) mice engrafted with tumours cells from humans with the proposed orphan conditions were the most common models used in the applications where non-clinical data was accepted to support MP. These models were also used in all submissions where only non-clinical in vivo data were used to support the MP criterion (*n* = 6; 17%).

### Data used to support SB at the initial OD stage

**Fig. 3:**
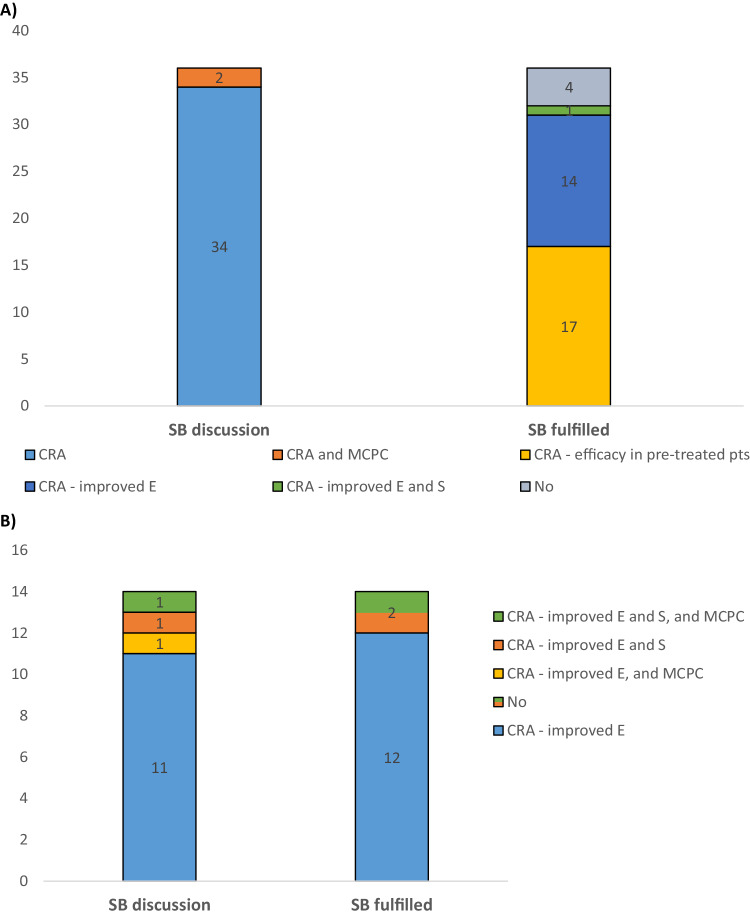
Arguments for SB (left) versus the claims accepted (right) to support the SB criterion in applications received for OD and maintenance of the status for genetically engineered ACT. The numbers of submissions were grouped in different coloured boxes according to the arguments given by the sponsors for SB and the claims which were accepted for fulfilling the SB criterion **A** at the initial OD stage (*N* = 36) and **B** at time of review of the criteria for maintenance of OD (*N* = 14). CRA clinically relevant advantage, E efficacy, MCPC major contribution to patient care, No SB criterion was not considered fulfilled, pts patients, S safety.

All 36 OD applications received for genetically engineered ACT in the period from 2014 to 2023 needed to show that the product applied for could be of SB to those affected by the orphan condition since other satisfactory methods for the proposed condition existed (Fig. 3A). It was noted that the arguments for SB were based on a CRA in 34/36 cases (94%), whereas in two of the submissions the sponsor claimed SB over existing therapies based on a CRA combined with major contribution to patient care. The same data used to support a potential efficacy of the proposed products were used to support an assumption of SB over existing therapies (Fig. 2). Of the 36 submissions for OD, 17 cases (47%) provided data that showed a clinically meaningful benefit in heavily pre-treated patients affected by the orphan condition, 14 (39%) submitted data which indicated improved efficacy over other satisfactory methods for the proposed condition, and 1 (3%) presented data supporting an assumption of both improved efficacy and better safety of the product. The claim of major contribution to patient care was not accepted in any of the two submissions where this was argued by the sponsor, but for one of the applications the claim of SB was accepted based on a CRA in terms of improved efficacy. In total, the sponsors of 4 (11%) of the 36 OD applications submitted failed to provide sufficient data to fulfil the SB criterion. The reasons for these failures were related to insufficient data to support an assumption of SB (1) over other authorised ATMP/innovative therapies demonstrated to be efficacious for the treatment of the proposed condition (i.e., DLBCL; 1 application), (2) limited clinical data (2 applications), or (3) availability of only in vitro data with the proposed product (1 application).

### Demonstration of SB at time of review of the criteria for maintenance of OD

At the stage of MA and review of the OD criteria, all 14 submissions received for the maintenance of the OD needed to provide data to demonstrate SB as there were at least one and up to six other satisfactory methods approved for the proposed therapeutic indications. Notably, for nine (64%) of these cases, the treatment landscape for the designated orphan condition had changed as one or more other medicines had been approved for the condition since the OD had been granted. In line with the OD applications, the arguments for SB of these products were primarily based on a claim of a CRA in terms of improved efficacy over existing therapies (Fig. 3B). This claim was accepted in most of the cases (12/14; 86%), but in two applications SB was not considered established based on the data presented.

**Fig. 4:**
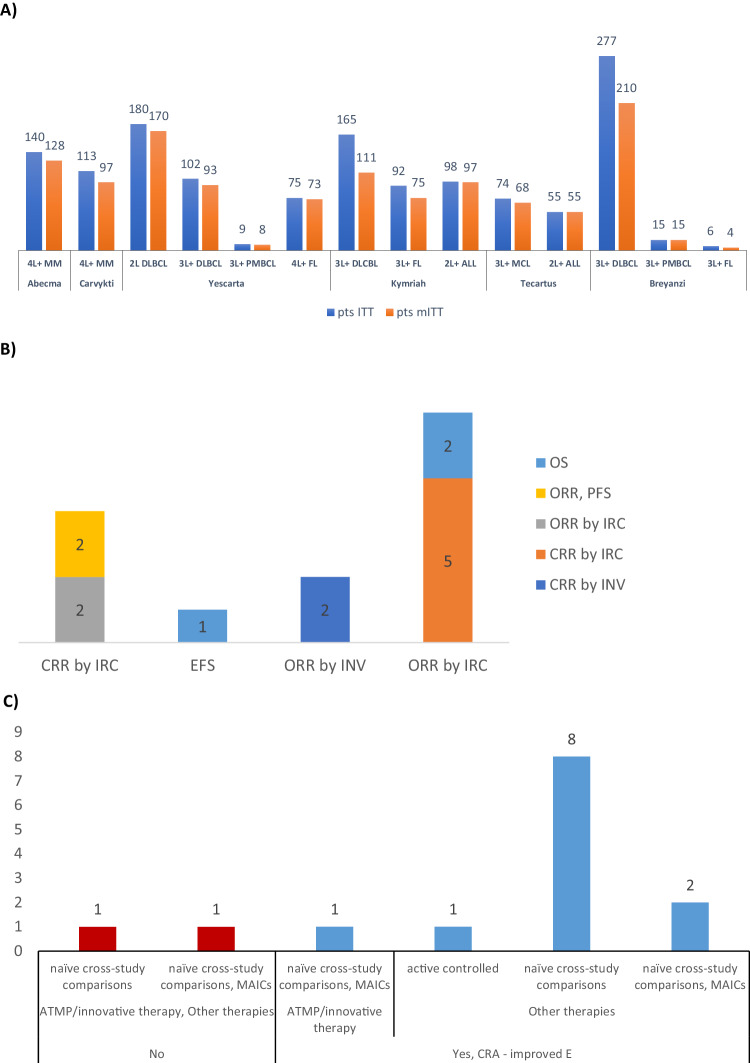
Clinical data used to demonstrate SB in applications for maintenance of the OD at time of MA or MA extension (*N* = 14). **A** Overview of numbers of patients and patient populations evaluated in the clinical data derived from the sponsors’ own studies of the new products which were used to justify SB. **B** The primary- (x-axis) and secondary endpoints (y-axis) of the clinical studies conducted by the sponsors used to support SB. **C** Numbers of submissions highlighting the type of comparative data presented and those accepted for demonstrating SB. “No” denotes those two cases where SB was not considered fulfilled. ALL acute lymphoblastic leukaemia, CRA clinically relevant advantage, CRR complete response rate, DLBCL diffuse large B-cell lymphoma, EFS event-free survival, FL follicular lymphoma, INV investigator, IRC independent review committee, ITT intent-to-treat (patients enroled), MCL mantle cell lymphoma, mITT modified ITT (patients infused/treated), MM multiple myeloma, ORR objective/overall response rate, OS overall survival, PFS progression-free survival, PMBCL Primary mediastinal large B-cell lymphoma, pts patients, 2 L second-line therapy, 2 L+ second- and later lines of therapy, 3 L+ third- and later lines of therapy, 4 L+ fourth- and later lines of therapy.

The clinical data used to confirm SB in the applications for maintenance of the OD were derived from the sponsors’ own clinical studies with their CAR-T cell products and mainly from single-arm phase 1 (*n* = 3), phase 1/2 (*n* = 2), or phase 2 (*n* = 8) studies. Only one of the applications which represented an indication extension to an earlier treatment line presented comparative data with the orphan medicinal product from a randomised controled phase 3 study. Most of the data with the proposed products were collected from relatively small patient populations and a few were based on observations from only a limited number of patients with the designated orphan condition (Fig. 4A). The arguments for SB were mainly based on results from a primary endpoint of either ORR or CRR and represented 93% (13/14) of the applications for maintenance of OD (Fig. 4B). The duration of these responses to the new products was considered a key secondary endpoint for demonstrating SB in all these 13 cases. The data used to support SB from the phase 3 study was based on the time-dependent primary endpoint of event-free survival, which was supported by the key secondary endpoint of overall survival.

The study results from the pivotal single-arm studies were contextualised with published clinical study data as external controls for the satisfactory methods of treatment for the target patient populations to substantiate the SB claim (Fig. 4C). Most submissions included naïve cross-study comparisons, with four also including matching-adjusted indirect comparisons (MAIC) to support a claim for SB of their product. Real-world data from retrospective studies and systematic literature reviews were also submitted in nine of the cases to further contextualise the clinical outcomes reported from the pivotal studies of the new products. In only four of these cases were real-world evidence regarded as sufficient to establish some of the claims for SB, whereas in other four cases they were only used as supporting evidence. One submission which included data from the phase 3 study used the active controlled comparison. Twelve of the 14 submissions were concluded to have provided sufficient data to demonstrate SB, whereas two failed to show a CRA over another approved CAR-T cell product.

Three of 14 submissions were withdrawn before an opinion was adopted and were based on data from one single-arm phase 1 study with the same CAR-T cell product, which was conducted in patients with three designated orphan conditions. It was noted that this product offered a SB for one of the designated orphan conditions, but that the sponsor failed to provide sufficient data to support the claim of SB for the other two conditions. The sponsor decided to withdraw all three OD to stay within the same global MA.

## Discussion

The results of this European regulatory landscape analysis of applications for OD and maintenance of the status for genetically engineered ACT over the last decade underscores the dynamic nature of these products in the treatment of rare cancers. Especially autologous CAR-T cell therapies play a significant role in treating rare haematological malignancies, predominantly targeting B-cells, with CD19 being the most frequently targeted antigen. The focus on CD19 for 7 different products, 6 of which obtained a total of 17 different OD, reflects its widespread expression on malignant B-cells and the efficacy of CD19-directed CAR-T cell therapies.

The finding that most of the products assessed by the COMP contained CAR-engineered T-cells (*n* = 19) and to a lesser extent TCR-modified T-cells (*n* = 4), reflects the CAR-T cell products prominence in the field of oncology due to their tumour antigen specificity. It has been highlighted in the literature that TCR-modified T-cells are not tumour antigen specific enough contrary to engineered CAR-T cells which have achieved a high level of tumour antigen specificity, particularly in haematological malignancies [3]. This specificity, coupled with the evolution of CAR-T cell engineering, has led to enhanced treatment efficacy compared to other authorised medicines.

The delivery of foreign gene material into human T-cells, a crucial step in engineered ACT, is accomplished using both viral- and non-viral vectors [8]. These viral vectors include retrovirus, lentivirus, adenovirus, and adeno-associated virus [21]. The COMP has predominantly observed the use of lentiviral vectors for the CAR-T cell products, aligning with their recognition as optimal tools for safe and effective gene transfer [22]. TCR-based therapy requires a structured and integrated process that involves patient screening for HLA subtypes in addition to the complex procedure required for autologous cell therapies (leukapheresis, generation of transduced product, lymphodepletion, and infusion of the final product) [23]. Although TCR-based therapy submissions were fewer, this strategy potentially has a broader applicability in targeting tumour-specific sequences presented in the major histocompatibility complex (MHC), which are inaccessible to CAR-based approaches [6, 24].

The regulatory review process revealed interesting trends and challenges. Of the initial OD applications received typically early during development, a high proportion were supported by preliminary clinical data from a limited number of patients with the target orphan conditions, indicating a strong reliance on clinical evidence for showing MP and efficacy of these products. Moreover, 89% of these cases contained data that successfully supported an assumption of SB of the new products based on a CRA over existing treatments, emphasising the therapeutic impact of these therapies.

The data supporting the potential benefit of a candidate product over authorised treatments is crucial for the decision-making when evaluating OD applications. The evidence can generally be based on non-clinical in vitro, in vivo and/or preliminary clinical data. In the case of genetically engineered ACT seen at the initial OD stage, around 80% included relevant clinical data which is higher than the range between 68 and 70% reported for all OD submissions assessed by the COMP [25]. The low proportions of submissions which were based only on non-clinical in vivo (17%) or in vitro data (3%) differs consequently from the overall OD applications received for all types of substances for which the COMP often will approve MP based on functional non-clinical in vivo data [25]. This may reflect the added complexity of non-clinical in vivo models considered valid for investigating CAR-T cell products. The only suitable murine models for studying potential efficacy of these types of products are humanised immunodeficient mice, such as NSG mice, since they are tolerant to and hence will not reject the human tumour xenografts or the human T-cell products. In accordance with this, all OD submissions based only on non-clinical in vivo data with the proposed product presented data from tumour xenograft models using humanised NSG mice.

Since authorised treatments for the proposed conditions were available, the criterion of SB had to be discussed for all procedures analysed which is a higher proportion compared to OD applications in general [26]. The COMP concluded either on a SB based on a CRA in pre-treated patients (often relapsed or refractory) or concluded on improved efficacy compared to other authorised treatment options. These findings are in line with the fact that the use of CAR-T cell therapies initially have been studied as last line of treatment. Indeed, it has been stated that CAR-T cell products have become an effective therapeutic option for patients with refractory blood cancers which points to the promising potential of this treatment approach [24, 27].

Of the genetically engineered ACT which were granted an OD, only six CAR-T cell products have reached the stage of MA. At this point, the criterion of SB must be confirmed with sufficient and robust clinical data from the development of the product [19], and it is required that the new product is put into context with existing treatment options. In view that most CAR-T products were initially studied in patients from single-arm phase 1 or phase 2 studies, the justification of SB was limited to indirect comparisons between the sponsor’s product and the satisfactory methods of treatment for the target patient population [16]. The sponsors predominantly submitted naïve cross-study comparisons and only four included a formal indirect comparison using MAIC. MAIC is a statistical method used to improve the robustness of indirect comparisons. By reweighting individual patient-level data from one study to the baseline summary characteristics of another, considering mutually reported treatment effect modifiers and prognostic factors, greater adjustment for relevant differences in population characteristics compared to naïve cross-study comparisons is aimed at and in the best case achieved [28]. However, challenges remain in conducting these comparisons because of issues like lack of access to individual patient-level data from the comparator studies, small patient numbers, differences in reporting, and methodological complexities such as inconsistencies of outcome definition and imbalances in important baseline characteristics after weighting. Still, the MAIC provided in 3 of these 4 cases helped the COMP in the assessment process and in determining a final opinion on the SB criterion. The COMP has highlighted the drawbacks of the indirect comparisons in the respective orphan maintenance assessment reports (OMAR) published on the EMA website for all authorised orphan medicines.

Five of the six CAR-T cell products which have been granted several OD and reached the stage of MA provided sufficient data in their submissions to demonstrate SB over existing satisfactory methods within the approved therapeutic indications. Eleven of the fourteen OD reviewed for maintenance at the MA stage therefore successfully obtained 10-year market exclusivities for their designated orphan condition, which protects them against competition from similar products within the same therapeutic indication. This is a major incentive in the EU Orphan Regulation.

In general, the COMP noted that the clinical data submitted was of high quality at both stages of assessment for engineered ACT. SB discussions were however complex due to the existence of other authorised treatments and treatment algorithms with several lines of therapy. The COMP has also noted that emerging treatments in haematological malignancies is a very dynamic field with some of these products even moving from last line to earlier treatment lines. A clear example is the rapidly evolving treatment algorithm for multiple myeloma for which there are over 20 products authorised and the therapeutic field for the management is continually changing. This rapid development is considered a positive trend, as these medicines are offering a new and innovative approach with the recent breakthrough in haematological malignancies changing the therapeutic approach to oncology [27, 29]. From a regulatory perspective and in the context of the decision-making regarding OD, new challenges emerge as it may be very difficult for new therapies to demonstrate that they offer a SB for patients affected by the disease under discussion when there are many authorised treatment options. Consistent with this, the failure to show SB when reviewing the OD criteria at the MA stage for two of the cases was related to a lower reported efficacy of the new product compared to another more effective CAR-T cell product approved for the target patient population. Therefore, the current evolving scenario requires active surveillance by product developers and regulators to bring effective treatment options to patients suffering from rare cancers.

In conclusion, the COMP’s decade-long review of applications for OD and maintenance of the status at the MA stage for genetically engineered ACT, especially CAR-T cell therapies, emphasises the growing importance of these treatments in rare haematological malignancies. The data reflect both the promise of these innovative therapies and the regulatory challenges in demonstrating their SB, especially when there are already several authorised medicinal products available to the target patient populations. The evolving nature of this field, with therapies moving from last line to earlier lines of treatment, underscores the need for ongoing vigilance and adaptation in both clinical and regulatory strategies to ensure further advancement.

## Data Availability

The datasets generated and analysed during the current regulatory analysis are available from the corresponding author on reasonable request.
